# Anal HPV in Kidney Transplant Recipients: A Closer Look at Pathogenesis and Clinical Management

**DOI:** 10.3390/pathogens15080848

**Published:** 2026-08-14

**Authors:** Sonia Moretti, Maria Rosaria Pavone Cossut, Annalisa Tiberi, Renato Pietroletti, Vittorio Unfer

**Affiliations:** 1 National Institute of Health, 00161 Rome, Italy; 2 Department of Obstetrics and Gynecology, San Salvatore Hospital, 67100 L’Aquila, Italy; 3 Department of Applied Clinical and Biotechnological Sciences, University of L’Aquila Surgical Coloproctology Hospital Val Vibrata, 64027 Sant’Omero, Italy; 4 Department of Gynecology and Obstetrics, UniCamillus—Saint Camillus International University of Health Sciences, 00131 Rome, Italy

**Keywords:** HPV, kidney transplant recipients, anal, ASCC, EGCG

## Abstract

Kidney transplant recipients (KTRs) face a significantly elevated risk of persistent high-risk human papillomavirus (HR-HPV) infection and subsequent anal squamous cell carcinoma (ASCC) due to chronic immunosuppressive therapy impairing immunosurveillance. Despite the markedly increased risk of ASCC in this vulnerable cohort, standardized screening protocols and optimal clinical management guidelines are not exhaustive. Chronically compromised immunosurveillance permits prolonged HR-HPV carriage and viral genome integration, driving oncogenesis via the E6 and E7 oncoproteins. While calcineurin inhibitors exert pro-oncogenic effects, transitioning to mTOR inhibitors offers distinct antiproliferative and antiviral advantages. Early detection relies on risk-stratified screening utilizing digital anorectal examination, anal cytology, and high-resolution anoscopy. For managing anal intraepithelial neoplasia, traditional topical agents and minimally invasive ablative procedures are widely used, although high recurrence rates present a major clinical challenge. This review analyzes HPV pathogenesis and clinical management in KTRs, evaluating the impact of immunosuppressive regimens and exploring innovative diagnostic and therapeutic strategies. To address viral persistence without compromising the allograft, integrating novel non-invasive, target-specific nutraceuticals may represent an interesting complementary approach. Ultimately, mitigating post-transplant ASCC requires a multidisciplinary strategy that couples early localized screening with tailored systemic immunosuppression.

## 1. Introduction

Human papillomavirus (HPV) is one of the most prevalent sexually transmitted infections worldwide. To date, more than 200 genotypes have been identified, approximately 40 of which infect the mucosal epithelium of the anogenital tract [1].

The natural cellular targets of HPV are keratinocytes, which constitute the stratified squamous epithelium of the skin and mucosal sites such as the anogenital tract and the upper respiratory tract. Different HPV types exhibit specific tissue tropism for either cutaneous or mucosal membranes across various body sites, displaying varying degrees of oncogenic potential [1,2].

HPV genotypes are classified into low-risk (LR-HPV) types, mainly associated with benign lesions such as anogenital warts, and high-risk HPV (HR-HPV) types, particularly HPV16 and HPV18, which are strongly associated with the development of intraepithelial neoplasia and invasive carcinoma [1,2,3].

In most immunocompetent individuals, HPV infections are transient and are either cleared or controlled within one to two years through effective innate and adaptive immune responses [1,4]. However, in approximately 20% of cases, HPV infection can persist, leading to severe clinical consequences that require strict monitoring and potential surgical intervention. Persistent HR-HPV infection is responsible for approximately 90% of anal cancers and practically all cases of cervical cancer [1,5]. Recent evidence also reported the potential utility of circulating HPV DNA biomarkers for optimizing the control of these virus-related cancers [6]. Given the crucial role of the immune system in clearing HPV infection, immunocompromised hosts are critically vulnerable to persistent infections. Furthermore, rapid disease progression toward high-grade squamous intraepithelial lesions (HSIL) and invasive cancer may occur in this population [7]. Among immunocompromised individuals, solid organ transplant recipients require long-term pharmacological immunosuppression, which may inadvertently facilitate HPV infection at anogenital sites [8,9,10]. Within this cohort, kidney transplant recipients (KTRs) exhibit the longest life expectancy and, consequently, the longest duration of immunosuppressive treatment. This lifelong regimen profoundly impairs antiviral T-cell-mediated immunity and reduces immunosurveillance against virally infected and dysplastic cells [8,11]. Such immunological impairment may facilitate the acquisition of different viral infections and malignancies [12,13]. Regarding HPV, immunocompromising therapies may contribute to both acquisition of new HPV infections and reactivation of latent infection, increasing the likelihood of persistent HR-HPV carriage and HPV-driven carcinogenesis [3,9,14]. Available evidence on the prevalence of anal HPV infection in KTRs remains limited. The largest comparative study reported an anal HR-HPV prevalence of about 45% among female KTRs and about 19% among male KTRs [15]. More recent prospective data revealed no statistically significant difference in the incidence of anal HR-HPV infection between KTRs and immunocompetent controls, while confirming a lower clearance and greater persistence of anal high-risk HPV infection among KTRs [5].

**Figure 1:**
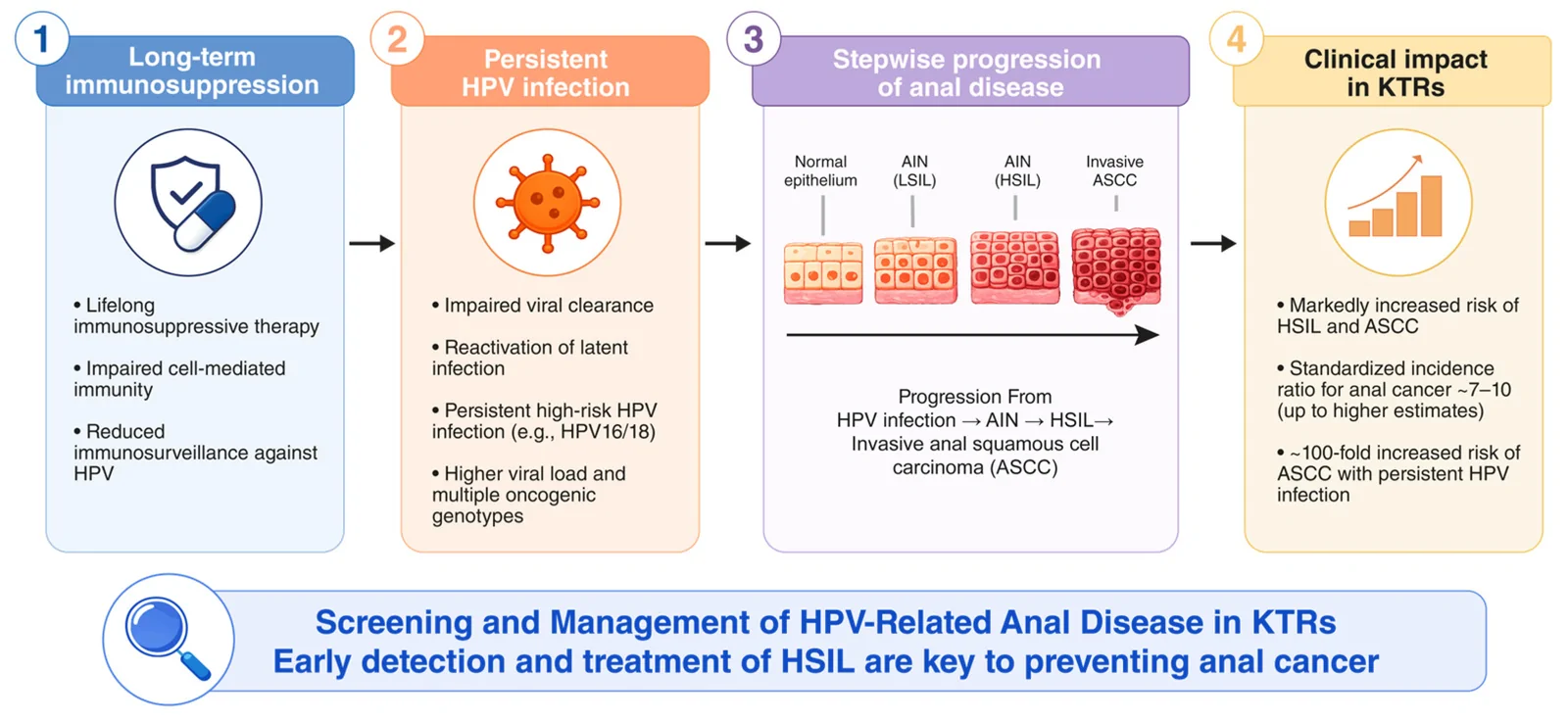
HPV-driven anal carcinogenesis in Kidney Transplant Recipients. Long-term immunosuppression impairs cell-mediated immunity and HPV immunosurveillance, favoring persistent high-risk HPV infection and viral reactivation. This promotes stepwise progression from normal epithelium to anal intraepithelial neoplasia (AIN), including low-grade squamous intraepithelial lesions (LSIL) and high-grade squamous intraepithelial lesions (HSIL), eventually leading to invasive anal squamous cell carcinoma (ASCC). As a result, kidney transplant recipients experience a substantially increased risk of HPV-related anal disease, underscoring the importance of screening and early treatment of precancerous lesions. Figure was generated by using Adobe Illustrator software.

Furthermore, cumulative exposure, intensity, and duration of immunosuppression are major contributors to susceptibility to dysplastic lesions, making pre-cancerous disease a key clinical concern in transplant medicine (Figure 1).

While the causal relationship between HPV and cervical malignancy is well established, accumulating evidence has also demonstrated a direct association between persistent HPV infection and anal cancer development. These findings emphasize the need to monitor this anatomical site and integrate screening and preventive strategies for high-risk populations. Given the increasing number of KTRs globally and their substantial long-term risk of HPV infection and HPV-related disease, a clearer understanding of anal HPV infection in this population is essential.

Accordingly, the aim of the present review is gathering current evidence on anal HPV infection in KTRs, with particular attention to the role of immunosuppression, and to explore innovative and alternative approaches for preventing and managing HPV-related anal disease.

## 2. Clinical Relevance of Anal HPV Infection in KTRs

Anal squamous cell carcinoma (ASCC) is the most prevalent type of anal cancer, accounting for approximately 80% of all anal cancers [16,17]. ASCC development is preceded by a stepwise progression from HR-HPV infection to anal intraepithelial neoplasia (AIN) and ultimately to HSIL, the recognized precursor lesion of invasive carcinoma (Figure 1).

Previous epidemiological studies have consistently demonstrated the increased burden of both anal precancerous lesions and ASCC related to HPV infection among transplant recipients. A systematic review and meta-analysis demonstrated a markedly increased incidence of ASCC in solid organ transplant recipients compared with the general population. This evidence confirms the substantial burden of anal HPV-related disease in this high-risk population [18].

KTRs exhibit a markedly increased incidence of anal precancerous lesions and subsequent malignancy compared with the general population. Large registry-based studies and systematic reviews have reported standardized incidence ratios for anal cancer ranging from approximately 7- to 10-fold higher, with even greater estimates depending on cohort characteristics and duration of post-transplant follow-up [18,19,20]. Persistent HR-HPV infection is considered the principal driver of anal carcinogenesis in KTRs, whose susceptibility to ASCC is further amplified by chronic immunosuppression, impaired viral clearance, and the prolonged persistence of oncogenic HPV infection [9,18]. Notably, considering the higher risk of immunocompromised individuals, KTRs appear to develop ASCC at a younger age than the general population, reflecting the long-term impact of chronic immunosuppression on HPV persistence and HPV-driven carcinogenesis [9]; this observation agrees with other findings in solid organ transplant recipients, where HPV-associated malignancies tend to occur earlier than in non-transplanted individuals [10].

Furthermore, epidemiological data indicate that the incidence of anal cancer increases progressively with time after transplantation, exceeding a 10-fold relative risk after approximately 10 years of follow-up. Together, these epidemiological findings support the adoption of long-term, risk-stratified surveillance strategies in KTRs. In particular, a crucial phase is during the late post-transplant period when the cumulative burden of chronic immunosuppression becomes more pronounced.

Specifically, a large retrospective Dutch single-center study retrospectively analyzed more than one thousand women renal transplant recipients who developed anogenital malignancies. Results demonstrated that the risk of developing ASCC was 122 times higher among this cohort of KTRs compared with the general population [21].

Despite this burden of anal carcinoma, comprehensive guidelines for anal cancer screening remain scarce. Because precancerous lesions precede invasive disease, early detection strategies are essential. The presence of HR-HPV genotypes, particularly HPV16, or abnormal cytology findings requires close monitoring through anal swabs, digital anorectal examination (DARE), and high-resolution anoscopy (HRA) with biopsy confirmation.

Clear recommendations regarding which populations should undergo routine screening, as well as standardized protocols for managing abnormal screening results, are currently lacking; a gap further exacerbated by limitations in available diagnostic tools.

Although the available evidence mainly derives from cervical HPV infection, it provides important insights that are also relevant to KTRs. Immunocompromised individuals are at increased risk of persistent HR-HPV infection, which promotes the development and progression of HPV-related dysplastic lesions. In women undergoing chronic immunosuppression, strict gynecological surveillance has been demonstrated to facilitate the early detection and treatment of cervical dysplasia, thereby reducing the burden of cervical cancer [22]. These findings reinforce the rationale for adopting similarly structured surveillance strategies in KTRs, who are likewise at increased risk of persistent HPV infection and HPV-associated anogenital malignancies because of long-term immunosuppressive therapy.

The rising incidence of anal cancer underscores the urgent need to establish risk-stratified screening recommendations. Because HPV-related ASCC develops through a stepwise progression from persistent infection to intraepithelial neoplasia and eventually invasive disease, preventive strategies targeting precancerous stages of carcinogenesis are attracting increasing clinical interest.

## 3. Insights into Pathogenetic Mechanisms

Lifelong immunosuppressive therapy in transplant recipients significantly elevates the risk of HPV-related anogenital pre-malignancies by altering the host–virus equilibrium. This therapy severely impairs cell-mediated immunity (CMI), specifically antigen presentation and HPV-specific CD4+ and CD8+ T-cell responses, thereby interfering with effective antiviral T-cell defense and diminishing tumor immunosurveillance [23,24,25].

Under physiological conditions, 80–90% of genital HPV infections clear spontaneously via robust Th1 and cytotoxic T-cell responses [1,4,8,26]. However, HR-HPV genotypes have evolved highly efficient immune evasion strategies that enable long-term persistence. These mechanisms involve suppression of antigen presentation pathways together with disruption of cytokine-mediated signaling processes. In addition, E7 has been reported to suppress activation of innate antiviral sensing pathways, including retinoic acid-inducible gene I (RIG-I) and the cyclic GMP-AMP synthase–stimulator of interferon genes (cGAS-STING) pathway. This consequently reduces interferon production and secretion and further impairs host antiviral responses [3,27]. Therefore, chronic inflammation can often accompany persistent infections, further contributing to tumor initiation and progression by promoting cellular survival and sustaining proliferative signaling pathways [28,29].

When long-term immunosuppressive therapy compromises this system, viral clearance fails [7,9,30]. This defect creates a tolerogenic microenvironment characterized by increased regulatory T-cell (Treg) activity and local immune exhaustion. As a result, transplant recipients experience high rates of new HPV acquisitions, reactivation of latent strains, higher viral loads, and persistent infection with multiple HR-HPV genotypes, which may clinically manifest as extensive treatment-refractory warts [7,9,14].

**Figure 2:**
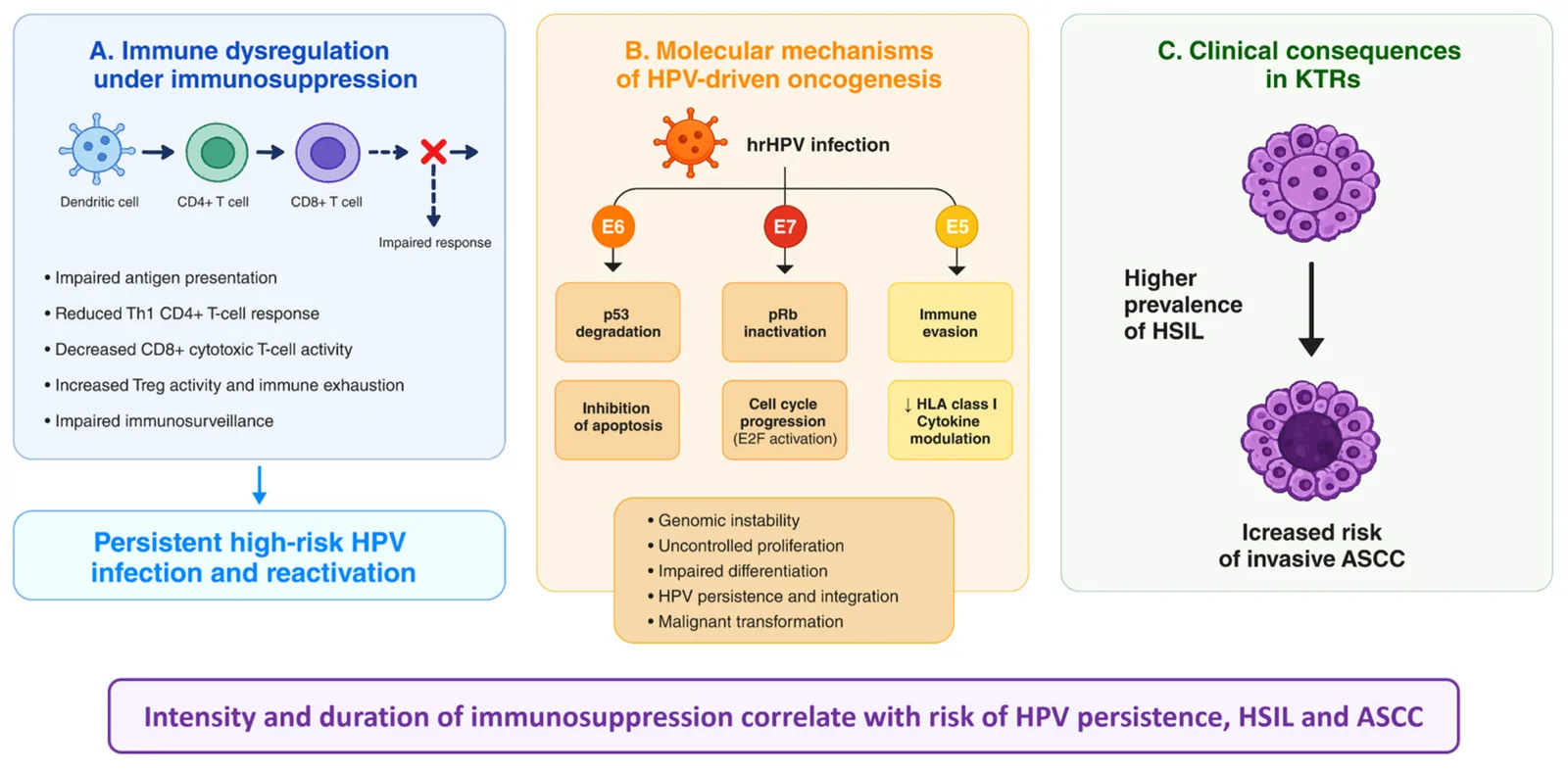
Immunopathogenesis and molecular mechanisms of HPV persistence and oncogenesis in Kidney Transplant Recipients. Immunosuppression disrupts antigen presentation and T-cell-mediated antiviral responses, facilitating persistent high-risk HPV infection and reactivation. At the molecular level, HPV oncoproteins E6, E7, and E5 promote carcinogenesis through p53 degradation, pRb inactivation, inhibition of apoptosis, immune evasion, and cytokine modulation. These alterations contribute to genomic instability, uncontrolled proliferation, impaired differentiation, viral persistence, and malignant transformation, ultimately increasing the prevalence of HSIL and the risk of invasive ASCC in KTRs. Figure was generated by using Adobe Illustrator software.

This compromised immunosurveillance permits prolonged HR-HPV carriage and viral genome integration within the epithelial basal layers, driving oncogenesis through the sustained expression of the E5, E6, and E7 viral oncoproteins (Figure 2) [31,32,33,34].

E7 binds and degrades the retinoblastoma protein (pRb), forcing S-phase re-entry and upregulating the biomarker p16 [35,36]. Concurrently, E6 induces the proteasomal degradation of p53 to suppress apoptosis, while modulating Hippo and JAK/STAT pathways to drive proliferation and inhibit cellular senescence [33,34,37]. Complementarily, E5 enhances EGFR signaling and downregulates HLA class I expression, shielding infected cells from remaining T-cell recognition [1,2,31,38]. Additionally, a functional interplay exists between p53, pRb, and the mTOR pathway, which HPV cancer cells utilize to evade senescence. The pharmacological approach based on mTOR inhibitors can effectively disrupt this mechanism [39,40]. Ultimately, this continuous, unmonitored oncoprotein activity explains why KTRs experience higher rates of persistent HR-HPV infection, HSIL, and an accelerated progression toward invasive ASCC [9,18,19].

Standard maintenance regimens typically combine corticosteroids, a calcineurin inhibitor (CNI), and either an antimetabolite or an mTOR inhibitor [24]. Among these, CNIs (cyclosporine and tacrolimus) exert a dose-dependent pro-oncogenic effect, where higher cumulative exposure directly correlates with increased cancer incidence. Corticosteroids at low doses do not independently elevate cancer risk, and data regarding mycophenolate mofetil (MMF) remains inconclusive and context dependent [24]. Notably, T-cell-depleting induction with anti-thymocyte globulin stands out as an independent risk factor for anogenital cancer [21,39].

Conversely, mTOR inhibitors (sirolimus and everolimus) exhibit distinct antineoplastic and antiviral properties. Converting from CNI-based regimens to mTOR inhibitors lowers overall cancer incidence and it is strongly encouraged upon malignancy development [24,39,40]. Mechanistically, they inhibit the PI3k/Akt/mTOR pathway, an upstream pathway crucial for viral replication that is activated by HPV and various herpes viruses [39,40]. Consequently, modifying the immunosuppressive regimen through CNI minimization and selective conversion to mTOR inhibitors can serve as a critical preventive strategy to reduce post-transplant malignancy risk in susceptible patients.

In conclusion, regardless of the specific drug strategy, there is consensus that transplant recipients face a higher long-term risk of *de novo* cancer due to extended life expectancy under immunosuppression. The increased incidence of ASCC over prolonged post-transplant periods confirms that deficient immunosurveillance sustains carcinogenesis during persistent HPV infection [9,19,39]. Therefore, modifying and tailoring immunosuppressive therapy in KTRs represents a vital preventive strategy to mitigate long-term malignancy risks in oncologically susceptible patients.

## 4. Current Perspectives from Detection to Clinical Management

### 4.1. Screening Strategies

ASCC is preceded by precancerous lesions known as AIN, graded from AIN-1 (low-grade dysplasia) to AIN-2/3 (high-grade dysplasia) [41]. Following the Lower Anogenital Squamous Terminology (LAST) classification, these correspond to low-grade squamous intraepithelial lesions (LSIL: AIN-1) and high-grade SIL (HSIL: AIN-2/3) [42,43].

In KTRs, management is uniquely challenging and the prognosis is worse than in the general population across all malignancies. The disease course is frequently more aggressive, and therapeutic options are limited because of patients’ comorbidities and the critical risk of graft rejection or loss, if immunosuppression is minimized [9,44,45]. Consequently, preventive measures are paramount, and screening is strongly recommended to monitor AIN and prevent ASCC [46,47].

**Figure 3:**
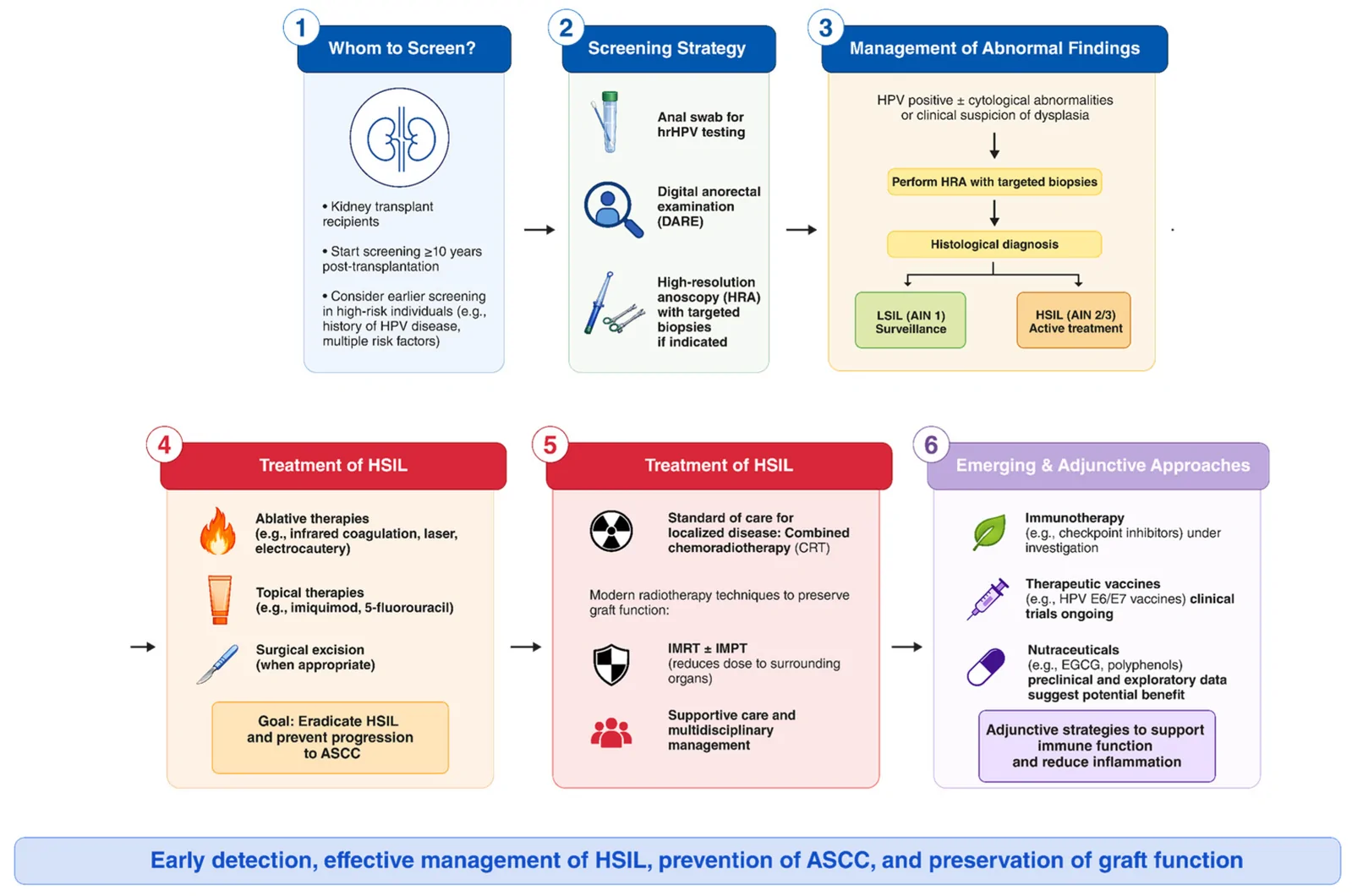
Screening and Management of HPV-related Anal disease in Kidney Transplant Recipients including therapeutic approaches. Recommended screening strategies include anal high-risk HPV testing, digital anorectal examination, and high-resolution anoscopy with targeted biopsies and the management of abnormal findings based on histologic diagnosis. Surveillance is recommended for LSIL/AIN1, whereas active treatment is indicated for HSIL/AIN2-3 to prevent progression to ASCC. Therapeutic options include ablative therapies, topical treatments, surgical excision in selected cases, chemoradiotherapy for localized invasive disease, and emerging adjunctive strategies under investigation. Emerging therapeutic strategies, including immunotherapy, therapeutic vaccines, adjunctive immunomodulatory interventions and nutraceuticals, may further support clinical management. Figure was generated by using Adobe Illustrator software.

The rationale for targeting HPV-related precancerous anal lesions stems from the biological similarities between the anus and cervix, the proven success of cervical cytology, and the clinical advantage of treating HSIL to decrease progression to invasive cancer [43]. To standardize practice, the International Anal Neoplasia Society (IANS) released consensus guidelines for anal cancer screening [48]. Current screening modalities include anal cytology (anal pap smear), HR-HPV testing (with HPV16 genotyping), or combinations of these tests. According to the IANS consensus guidelines, individuals with abnormal screening results or clinical suspicion should be referred for HRA, which represents the reference standard for the diagnosis and histologic confirmation of anal dysplasia [45,46,47,48,49]. These screening strategies are intended for settings with access to HRA. Where HRA is unavailable, DARE remains the recommended approach for detecting early anal cancer [46,50]. This limitation is critical, considering that diagnostic performance of HRA is significantly superior to standard proctological examination using conventional anoscopy [47] (Figure 3).

Because HSIL is the recognized precursor of ASCC, its early detection and treatment represent a cornerstone of preventive management in high-risk populations, including KTRs, with the aim of reducing progression to invasive disease [43,51,52].

Furthermore, the presence of HR-HPV genotypes, cytological abnormalities, or clinical suspicion of dysplasia, even in the absence of visible lesions within the anal canal, requires a close clinical follow-up [20]. In these scenarios, comprehensive monitoring via anal swabs, DARE, and HRA with targeted biopsies of suspicious areas is strongly indicated.

Following IANS guidelines, a proctological examination, including margin inspection, DARE, and standard anoscopy, is indicated for symptomatic individuals [43,48]. The DARE should be performed routinely to detect palpable early tumors, especially when HRA is unavailable, though it fails to identify non-palpable anal HSIL [43,48].

Anal smear testing consists of collecting anal canal transitional cells by a swab [53,54], preferably 48 h after intercourse and in the absence of active infections or treatments [53,54]. While its diagnostic performance in identifying ASC-US and more severe abnormalities is comparable to cervical screening [50], with accuracy increasing with patient age and lesion size/multiplicity, it has notable limitations [43]. Specifically, its low specificity can lead to unnecessary HRA procedures and the correlation between the severity of cytological abnormalities and histological findings remains relatively weak; although high-grade cytology strongly predicts histopathological HSIL, low-grade findings do not rule it out [50].

During screening analyses, PCR testing identifies HR-HPV genetic material from transitional zone cells [5,54,55]. Previous research indicates that while HR-HPV testing is highly effective in identifying HSILs, offering a high negative predictive value that reliably rules out dysplasia, its overall specificity remains limited in high-risk groups [50]. Focusing analysis to HPV16 detection rather than testing for the broader spectrum of HR-HPV genotypes enhances specificity at the expense of sensitivity [50]. Nonetheless, when compared to anal cytology, comprehensive HR-HPV testing demonstrates comparable sensitivity while yielding superior specificity [43].

Following an abnormal cytology result, HRA remains the gold standard for biopsy confirmation. Several studies have indicated that, although anal cytology is an effective screening tool, it may underestimate the severity of dysplastic lesions when compared with HRA-guided biopsy in KTRs [47,56]. This discrepancy limits the reliability of cytology as a standalone diagnostic approach and may lead to underestimation of the true disease burden. Furthermore, the agreement between cytology, histopathology, and conventional anoscopy is limited [57], whereas the combined use of anal cytology and HRA improves the detection of clinically significant lesions [47,58]. More recently, the integration of artificial intelligence into HRA has promised a supportive tool for improving the identification of suspicious areas requiring targeted biopsy [59].

Another important limitation is the imperfect correlation between cytological abnormalities and histopathological findings. In KTRs, HRA-guided biopsy may identify HSIL despite low-grade or even negative cytological findings, underscoring the limited sensitivity of anal cytology as a standalone screening modality [47,56,57]. Therefore, negative or low-grade cytological results should not be considered sufficient to exclude clinically significant disease in this high-risk population, and abnormal screening findings should prompt further evaluation with HRA whenever available, according to current consensus recommendations [47,48,50]. This underscores the importance of HRA as a complementary diagnostic modality, particularly in individuals with abnormal screening results or persistent HR-HPV infection, where the combination of cytology and HRA improves the detection of clinically significant lesions [47,58].

To overcome the suboptimal specificity of traditional screening tools, research focuses on novel biomarkers to improve risk stratification, diagnosis and prognostic assessment [43,60]. Among these biomarkers, HPV E6/E7 mRNA testing has attracted considerable interest as it reflects the active oncogene transcription, making it a more specific indicator of transforming infection than DNA testing. While the literature highlights its potential utility for detecting premalignant anal lesions and anal cancer, high study heterogeneity and the limited amount of evidence currently prevent its widespread clinical implementation [43,50,60].

Immunohistochemical biomarkers have also emerged as valuable adjunctive tools in evaluating anal intraepithelial lesions. Specifically, p16INK4a (p16) is overexpressed following pRb inactivation by the viral E7 protein, serving as a surrogate marker of transforming HR-HPV infection [61]. Because p16 expression correlates with lesion severity, it aids in diagnostic standardization and helps differentiate LSIL from HSIL in equivocal histopathological cases [61]. Furthermore, evaluating the co-expression of p16 (cell-cycle deregulation) and Ki-67 (proliferation) within the same cell improves HSIL identification, though current data regarding this combination remains limited and heterogeneous [62].

In recent years, epigenetic biomarkers have attracted growing interest, as altered host and viral DNA methylation patterns correlate with persistence and malignant progression. These molecular changes could potentially separate lesions at high risk from those likely to regress, though large prospective validation is still required before their incorporation into routine clinical practice [43]. In addition, circulating HPV DNA has recently emerged as a promising non-invasive biomarker for disease detection, prognostic assessment, and treatment monitoring in HPV-associated malignancies. Although its clinical application in anal disease, particularly in KTRs, remains to be established, it represents an area of growing research interest [6].

Ultimately, while these emerging biomarkers represent a great promise, they are not yet ready as standalone tools; future research must define their integration into existing screening and surveillance strategies for HPV-associated anal disease [48,60].

### 4.2. Therapeutic Approaches: Traditional and Innovative Strategies

Managing AIN in immunosuppressed patients, particularly KTRs, remains clinically challenging due to anatomical complexity, high HPV-related recurrence rate, and impaired immune surveillance from chronic immunosuppressive therapy.

Prophylactic HPV vaccines are highly effective in preventing HPV infection and HPV-related malignancies in immunocompetent individuals; therefore, they are recommended as part of routine vaccination programs [4]. However, solid organ transplant recipients, particularly KTRs, are at substantially increased risk of persistent high-risk HPV infection and HPV-related malignancies because of chronic immunosuppression [9,18]. Although currently available non-live HPV vaccines are considered safe in transplant recipients, vaccine immunogenicity is reduced when vaccination is administered after transplantation. Consequently, current clinical guidelines recommend completion of the full HPV vaccination schedule before transplantation whenever feasible in order to maximize vaccine-induced immunity [10,63]. Nevertheless, vaccination does not eliminate the need for long-term surveillance, and vaccinated KTRs should continue to undergo appropriate screening for HPV-related malignancies because of their persistently elevated oncological risk [63].

Treatment strategies are primarily guided by lesion grade, size, anatomical location, symptom burden, and the degree of immunosuppression. In general, low-grade lesions (AIN I) have limited malignant potential and are managed conservatively. However, in transplant recipients, even low-grade lesions may enlarge over time and report a greater tendency to progress. For this reason, treatment of AIN I may be considered for symptomatic relief, but also in selected high-risk patients. Conversely, high-grade lesions (AIN II and AIN III) are precursors of invasive anal carcinoma and require active treatment to reduce the risk of malignant transformation (Figure 3).

Several local therapeutic options are currently available, with topical treatments representing a minimally invasive approach suitable for multifocal or superficial lesions. Among these, imiquimod 5% cream is an immune response modifier drug that activates Toll-like receptor 7, leading to the production of pro-inflammatory cytokines, including interferon-α, tumor necrosis factor-α, and interleukin-12. In this way, it promotes a Th1-mediated antiviral immune response and enhances cell-mediated clearance of HPV-infected cells [64]. Clinical studies and systematic reviews have demonstrated that imiquimod can induce regression of anal intraepithelial lesions in selected patients. It acts by reducing HPV viral burden and decreasing the expression of p16, which suggests direct activity against HPV-infected dysplastic cells [64]. However, treatment response is variable, and lesion recurrence remains relatively common, highlighting the need for careful post-treatment surveillance [65]. Furthermore, while clinical studies have reported encouraging response rates, its use in managing AIN remains off label and its evidence in transplant recipients is still limited and adverse effects like local irritation, pain or inflammatory reactions may compromise treatment adherence. In KTRs, the use of topical therapies should be individualized, considering the degree of immunosuppression, lesion characteristics, and the potential for reduced therapeutic efficacy compared with immunocompetent individuals [63].

Another commonly used topical therapy is 5-fluorouracil (5-FU), an antimetabolite that interferes with DNA synthesis by inducing lesion regression. However, its regimens are not standardized, and local discomfort frequently requires temporary interruption. Additional topical agents include cidofovir, which exhibits in vitro antiviral activity against HPV [65], and trichloroacetic acid, an inexpensive, safe cytotoxic compound that destroys small, localized lesions via chemical coagulation of proteins [65].

For larger lesions or high-grade lesions, ablative procedures are generally preferred. Traditional surgical excision is now less favored due to significant morbidity, including postoperative pain, anal stenosis, incontinence, and impaired sexual function. Consequently, less invasive destructive techniques are increasingly used in clinical practice. Although evidence specifically addressing KTRs remains limited, infrared coagulation is considered a reasonable ablative option for appropriately selected patients. Indeed, it has a favorable safety profile and extensive experience in the management of anal intraepithelial neoplasia [65]. Photodynamic therapy achieves ablation of AIN by applying light sources to previously photosensitized areas [66], while HRA-guided electrocautery ablation allows targeted destruction of dysplastic lesions while preserving surrounding healthy mucosa. Other options include laser ablation, radiofrequency ablation, and argon plasma coagulation.

The management of HPV-related lesions in transplant recipients is further complicated by chronic immunosuppression, which fosters recurrent, multifocal, and treatment-refractory disease. In patients with extensive, asymptomatic lesions, some clinicians prefer close surveillance rather than immediate aggressive intervention to avoid cumulative tissue damage. At the same time, reducing the immunosuppressive burden should be considered when clinically feasible.

Modifying the immunosuppressive regimen is also an increasingly recognized strategy. While calcineurin inhibitors, like tacrolimus and cyclosporine, may increase long-term oncologic risk through potential pro-oncogenic properties, conversely mTOR inhibitors, such as sirolimus and everolimus, can reduce post-transplant malignancies due to their potential antiproliferative and antiviral properties.

Because persistent HPV infection is a major risk factor for oncogenic progression, managing viral persistence, even in the absence of symptomatic lesions, is crucial, carrying an even higher clinical relevance in immunocompromised patients. Indeed, in the absence of effective therapeutic intervention, persistent HPV infection may lead to the progressive development of premalignant lesions, which can eventually evolve into invasive carcinoma. The transition from precancerous lesions to malignant disease is driven by the gradual accumulation of additional genomic abnormalities, including both tumor suppressor gene inactivation and oncogene activation, ultimately culminating in the development of invasive cancer [29].

In this scenario, despite the availability of multiple local treatments, recurrence of the infection remains one of the greatest clinical challenges: completely eradicating persistent HPV infection is increasingly difficult, making long-term surveillance mandatory.

The emerging literature points to potential novel nutraceutical approaches to support clinical management of HPV persistent infection (Figure 3): they may specifically target viral persistence mechanisms rather than providing a generic immune boost [67,68,69]. Among the compounds investigated for their possible interference with the viral lifecycle, Epigallocatechin gallate (EGCG) has reported interesting results in modulating cellular pathways involved in viral replication and tumor progression [70,71]. Biologically, EGCG may effectively impede the oncogenic cycle of HPV by suppressing the activity of viral oncogenes E6 and E7, thereby restoring the function of the tumor suppressors p53 and pRb [72,73].

Along with EGCG, other natural molecules have reported a potential role in counteracting HPV infection and its persistence [71]. For instance, adequate systemic levels of folate and vitamin B12 appear crucial in this context: their deficiency is strongly linked to an increased susceptibility to persistent HR-HPV infections, as these vitamins interact to maintain optimal genomic DNA methylation and genomic stability, thereby reducing malignant progression [74]. Notably, since HPV entry is facilitated by mucosal tissue damage, molecules such as hyaluronic acid (HA) can support tissue repair and strengthen the genital and anal epithelial barrier, preventing the virus from exploiting micro-lesions to access basal cells and establishing a chronic infection [75,76].

More recently, clinical and preclinical studies have reported encouraging preliminary evidence supporting the use of the above-mentioned natural compounds as adjunctive strategies for HPV management. Although the primary references on these molecules focus on cervical HPV infection, the biological activity of the virus suggests that these findings may plausibly extend to HPV infections at other anatomical sites. However, since the available evidence specifically concerns the cervical site, it should be regarded only as a starting point—rather than definitive proof—for investigating HPV infection at, for instance, the anal site, which warrants dedicated future research.

Experimental investigations have demonstrated antiviral, antioxidant, immunomodulatory, and tissue-repair properties that may interfere with viral persistence and HPV-mediated cervical carcinogenesis [71,72,73,74,75,76]. In parallel, from a clinical perspective, studies have reported improvements in HPV clearance and regression of low-grade HPV-related epithelial lesions, particularly in women with cervical HPV infection [77,78,79]. Cohort clinical studies reported improved HPV DNA clearance and regression of low-grade cervical lesions (e.g., LSIL and ASCUS) within 12 to 24 weeks [78], with recent data reporting clearance rates up to 85.8% [79]. Interestingly, clinical cases reported the effectiveness of the oral supplementation with these natural compounds in a patient with a 3-year persistent anal HPV infection with a condylomatous lesion [77]. However, the current lack of randomized controlled studies underlines the preliminary nature of these findings, therefore further rigorous clinical trials are necessary to extend the applicability to other HPV-associated diseases and high-risk populations such as KTRs. While alternative dietary interventions are often based on general immune boosts, using molecules directly targeted to viral activity offers a more specific, evidence-based profile for non-invasive HPV management. Furthermore, given the specific molecular mechanism of action—which targets conserved viral pathways—it remains biologically plausible that these effects could extend to other anatomical sites. Nevertheless, further clinical studies remain essential to fully evaluate and support the role of these and other molecules in the management of this high-risk population, including KTRs.

## 5. Limitations of the Available Evidence

Some limitations of the evidence discussed should be acknowledged. Much of the available literature is based on retrospective, single-center studies with relatively small sample sizes, limiting statistical power and the generalizability of the findings. In addition, considerable heterogeneity exists across studies in terms of transplant populations, immunosuppressive regimens, and patient characteristics, making direct comparisons challenging. Variability in HPV testing methods, screening algorithms, and diagnostic approaches further contributes to inconsistencies in the reported prevalence of anal HPV infection and related lesions. Moreover, the available data on emerging nutraceutical approaches and therapeutic strategies mainly concern HPV infection at the cervical site. Although the molecules involved act on viral mechanisms that are not strictly site-specific—suggesting their activity could extend to the anal site as well—this distinction should be kept in mind, and the current evidence should be regarded as a starting point for future research rather than definitive proof. Additionally, KTRs represent a particular population of immunocompromised individuals, whose specific characteristics warrant further dedicated investigation.

## 6. Methods

We searched through the principal biomedical databases, including PubMed/MEDLINE, Scopus, and Web of Science, for studies published in English up to June 2026. The literature search focused on publications addressing the pathogenesis, screening, diagnosis, prevention, and management of anal HPV infection in KTRs. The search strategy included the following keywords, used both individually and in combination: kidney transplant recipients, renal transplant recipients, human papillomavirus, HPV, anal HPV infection, immunocompromised patients, immunosuppression, anal cancer screening, anal cytology, HPV vaccination, natural molecules.

## 7. Conclusions

Eradicating persistent anal HPV infection and preventing subsequent ASCC remains challenging, especially in vulnerable populations such as transplant recipients, particularly KTRs. Indeed, in such populations long-term surveillance stands as an indispensable cornerstone of clinical management. The intersection of chronic, life-long immunosuppression with the aggressive oncogenic mechanisms driven by E6 and E7 oncoproteins demands a shift from reactive management to proactive, standardized clinical protocols.

Looking forward, enhancing diagnostic accuracy, alongside novel complementary therapies and future HPV-specific immunotherapies, paves the way for promising multidisciplinary approaches that balance local interventions with systemic immune factors. To this end, this review aimed to bring this highly at-risk population into sharp focus, systematically evaluating the current screening strategies and management options available.

## Data Availability

No new data were created or analyzed in this study. Data sharing is not applicable to this article.
